# On-ground compliance with tobacco control law by Central Universities in India

**DOI:** 10.18332/tpc/183682

**Published:** 2024-02-12

**Authors:** Raja Singh

**Affiliations:** 1 Department of Architecture, School of Planning and Architecture, New Delhi, India; 2 Built Environment and Public Health Research Fellow, Tathatara Foundation, Vizianagaram, India; 3 ISAC Centre for Built Environment Policy, Mysore, India

**Keywords:** anti-smoking law, anti-tobacco law, educational institutions, India, young people, right to information

**Dear Editor,**

India created a tobacco control law in 2003^1^. One of the main focuses of this law was young people, as the law was extended for compliance in educational institutes. As young people attended educational institutes, the sale of cigarettes and tobacco within 100 yards of educational institutes was prohibited by law, and every educational institute had to put up a signboard stating the same, at a conspicuous point outside the premises^2^. The Indian Government further banned smoking in public places, with a unique exercise of decentralizing the power to penalize the offenders in India^3^. This means that instead of law enforcement agencies, the station masters in railway stations, postmasters in post offices, and librarians in libraries could now compound the offense and collect the fines. This also meant that heads of educational institutes/principals/teachers now had the legal responsibility to enforce the tobacco control law made by the Indian legislature to prohibit smoking in public places. This also means that the actual implementation of the anti-smoking law, with respect to young people, would vest in educational institutes, and the metrics of compliance from these would considerably reflect upon the compliance of the tobacco control law in India. To check the same, applications for information under India’s transparency law, the Right to Information Act 2005^4^, were filed with 22 Central Universities in India. These are the premier higher education institutions in the country. Out of the 40 central universities^5^, 21 would represent a confidence level of 90 ± 12% margin of error. The status of law implementation in the centrally supported, public-funded universities designated as ‘Central Universities’ represents the situation across other premier or major universities in the rest of the country (excluding some rural or peripheral universities).

For this study, applications under the Right to Information Act 2005 were filed requesting information from the 22 Central Universities. The parameters on which the information was sought were based on the Indian tobacco control law, and points related to universities were selected after reading the complete law, inclusive of amendments and rules. The information sought was in the format as worded in Table 1, where the results are presented after compilation. It was seen that there is no universal compliance with the anti-smoking/tobacco legislation. The fine collection compliance was only 41%, and the boards’ compliance was 36.4–72.8%. This needs to be highlighted for four reasons. The first is the fact that these, as educational institutions, have been provided decentralized power by statute to implement the anti-smoking/tobacco regime. The second important factor is that there exists smoking and tobacco use by the youth, specifically in these institutions, and tobacco control is needed in these. Third, most importantly, these Central Universities being centrally funded may be the perceptional reference points for other universities and institutes in rural areas and the peripheral areas. Lastly, the important step in compliance requires a simple intervention of putting up signboards and collecting fines.

**Table 1:** The information from 22 Central Universities was sought by an application under the Right to Information Act 2005. The parameters based on which information was collected were derived from the tobacco control law and its various amendments and rules. These were all related to educational institutes, as applicable to Central Universities. Out of the 40 central universities, 22 included in the study represent a confidence level of 90 ± 12% margin of error. The information was collected in 2023 from the 22 Central Universities, which are funded by the Central Government of India

*Compliance parameters*	*Full Compliance n (%)*	*Partial Compliance n (%)*	*No Compliance n (%)*	*Did not supply information n (%)*	*Remarks*
Whether the board stating ‘Prohibition of sale of cigarettes and other tobacco products within 100 yards of the educational institution’ was installed at a conspicuous place [2]	8 (36.4)	2 (9.0)	5 (22.8)	7 (31.8)	2 out of the 5 Universities with No Compliance (or no board installed) stated that they had initiated the process of installation.
Whether the ‘No Smoking Area – smoking here is an offence’ board has been installed at various places on the campus [3]	16 (72.8)	0 (0)	2 (9.0)	4 (18.2)	It is mandatory that No Smoking boards be installed in all public places, including educational institutions.
Whether instances of smoking have been recorded and a fine has been collected/warnings issued	9 (41.0) Institutes with fines collected: 1; Institutes with no instances/no fines: 8	-	-	13 (59.0)	The Head of the Institution/Principal/Headmaster/Teacher has been given the power to compound offences and collect fines. See Schedule III of The Prohibition of Smoking in Public Places Rules, 2008 [3]
Whether the institute has the presence of cigarette and tobacco vendors within 100 yards	10 (45.5) No vendors within 100 yards	12 (54.5) Information not on record	-	-	As this information record may not be under the university’s responsibility, the proactive universities have provided information. The ones with no record will not be considered as denying information.
Whether any activity/event/ circular/initiative to raise awareness on the prevention of the use of tobacco and smoking, has been done by the institutes	17 (77.3)	-	-	5 (22.7)	This is not a statutory requirement. Prevention awareness ranges from issuing circulars/events/celebrating ‘No Tobacco Day’, etc.

Full compliance: the tobacco control law was followed to the letter in spirit. Partial compliance: the law was not fully complied with, but part of the step was done, e.g. the board was present, but with incorrect wording or using metres instead of yards to denote distance. No compliance: the tobacco control law was not followed, neither fully nor partially. Information not provided: as the information was requested through the Right to Information Act 2005, the universities that denied the information are the ones mentioned here.

There are multiple previous studies that deal with a part of the compliance of the anti-smoking/anti-tobacco law of India, but most either have a focus on the presence of vendors around the institutes, or they may engage in the evaluation of the socio-behavioral aspect of tobacco use and smoking^6-10^. This study is unique as it lists all the compliances, including signboards of two different formats and collection of fines, the latter being not studied before.

If India has to bring about control of the youth smoking epidemic or implement a well-meaning anti-smoking/anti-tobacco law, the first step must be compliance by educational institutions where young people study. The Central Government institutes and designated Central Universities should take a positive lead.

## Data Availability

This study has used information in the public domain.
